# Canine Olfactory Thresholds to Amyl Acetate in a Biomedical Detection Scenario

**DOI:** 10.3389/fvets.2018.00345

**Published:** 2019-01-22

**Authors:** Astrid R. Concha, Claire M. Guest, Rob Harris, Thomas W. Pike, Alexandre Feugier, Helen Zulch, Daniel S. Mills

**Affiliations:** ^1^Animal Scent Detection Consultancy and Research, Santiago, Chile; ^2^School of Life Sciences, University of Lincoln, Lincoln, United Kingdom; ^3^Medical Detection Dogs, Milton Keynes, United Kingdom; ^4^Royal Canin SAS, Aimargues, France; ^5^Dog Trust, London, United Kingdom

**Keywords:** olfactory thresholds, amyl acetate, detection, accuracy, sensitivity

## Abstract

Dogs' abilities to respond to concentrations of odorant molecules are generally deemed superior to electronic sensors. This sensitivity has been used traditionally in many areas; but is a more recent innovation within the medical field. As a bio-detection sensor for human diseases such as cancer and infections, dogs often need to detect volatile organic compounds in bodily fluids such as urine and blood. Although the limits of olfactory sensitivity in dogs have been studied since the 1960s, there is a gap in our knowledge concerning these limits in relation to the concentration of odorants presented in a fluid phase. Therefore, the aim of this study was to estimate olfactory detection thresholds to an inert substance, amyl acetate presented in a liquid phase. Ten dogs were trained in a “Go/No go” single scent-detection task using an eight-choice carousel apparatus. They were trained to respond to the presence of solutions of amyl acetate diluted to varying degrees in mineral oil by sitting in front of the positive sample, and not responding to the 7 other control samples. Training and testing took place in an indoor room with the same handler throughout using a food reward. After 30 weeks of training, using a forward chaining technique, dogs were tested for their sensitivity. The handler did not assist the dog during the search and was blind to the concentration of amyl acetate tested and the position of the target in the carousel. The global olfactory threshold trend for each dog was estimated by fitting a least-squares logistic curve to the association between the proportion of true positives and amyl acetate concentration. Results show an olfactory detection threshold for fluid mixtures ranging from 40 parts per billion to 1.5 parts per trillion. There was considerable inter-dog difference in sensitivity, even though all dogs were trained in the same way and worked without the assistance of the handler. This variation highlights factors to be considered in future work assessing olfactory detection performance by dogs.

## Introduction

The olfactory abilities of dogs are widely documented in the literature and are generally thought to be superior to currently available man-made sensors (1–6). Accordingly, dogs are used worldwide in a variety of chemical detection tasks for civilian, military, wildlife, and medical detection purposes [e.g., (7–10)]. Despite their importance as biological sensors protecting life and property, relatively little research has focused on the measurement of the limits of the dog's olfactory sensitivity. The olfactory detection threshold, [ODT, (11)] is the minimum concentration of an odorant stimulus an individual is able to reliably detect and differentiate from a blank sample (12–15), and may be defined, alternatively, in terms of a performance criterion relating to a detection task (e.g., percent of correct responses/true positives) (16, 17). The dog's olfactory threshold has been estimated as being within the parts-per-billion (ppb) to parts-per-trillion (ppt) range for a variety of chemical odors. For example, Moulton et al. (18) reported a detection threshold for aliphatic acids such as propionic acid at 10,000 ppm and acetic acid at 100,000 ppm; by contrast, Marshall et al. (17) determined a threshold for n-pentanoic acid of between 1 and 100 ppb using the performance criterion of a 50% correct response. The detection threshold for more complex chemical odors such as methyl benzoate, cyclohexanone, and nitroglycerin has been determined to be between 0.1 and 10 ppb (12, 19). Although data derived from laboratory studies are expected to provide substantial information about olfactory sensitivity, determinations may be unreliable or lack reproducibility. A major issue for assessing the threshold levels reported by different studies is that varied methodologies have been used, which gives rise to very different threshold estimations for the same odors (19–21), even when performed by the same investigators (18). For instance, (22) using a conditioned suppression paradigm to determine the dog's olfactory sensitivity to amyl acetate in six Beagles, reported it to be between 52 and 32,600 ppt, while (23) observed a positive spontaneous electroencephalographic olfactometry response only at a threshold concentration of 1 ppm in six Beagles. Finally, (24) trained two dogs (Standard Schnauzer and Rottweiler) in field conditions to recognize n-amyl acetate in retriever tubes and then, tested them using a chamber box. This resulted in detection values of 1.9 and 1.14 ppt. According to the authors, training methods based on positive reinforcement, non–restrained conditions and a more natural search scenario, were the main reasons for the much higher sensitivity, roughly 30–20,000 times lower than the thresholds reported in previous studies produced by more conventional laboratory procedures (e.g., using water deprivation and punishment) (17, 18, 22).

Over the past decade, dogs have been widely trained to work under controlled laboratory settings to check different samples and discriminate between target (i.e., the conditioned odor) and non-target samples using a reward-based approach (i.e., food or toy rewards) and non–restrictive searching systems, such as multi-choice apparatus and line-ups [e.g., (2, 25–27)]. In these non-restrictive searching systems, the samples with different odors are placed next to each other in a straight line-up or a circular one (carousel) and the dog has to identify the target sample by showing a trained alert response, and ignore the non-target samples. Scent detection tasks performed by dogs in a laboratory environment have involved forensic human scent match-to-sample tasks (28, 29) and diagnostic procedures for biomedical applications (8, 30). In a biomedical detection scenario, dogs detect disease biomarkers in human samples, which may relate to a particular cancer, bacterial or viral infections [e.g., (3, 30–33)]. As a biomedical detection sensor for human diseases, dogs can be trained to detect volatile organic compounds (VOCs) in low concentrations that might range from parts per million or even parts per trillion. The metabolism of infected cells slightly changes the odor of these VOCs compared to those of someone who is healthy (34–36) and so unique, chemical compositions are naturally emitted into the blood and bodily fluids when someone has a disease. Potential volatile organic compounds biomarker concentrations are reported to be in the range of parts per billion in blood and urine (34), which may be detected by dogs with a high degree of olfactory acuity. Although VOC biomarkers appear to be within the potential detection range of a dog's olfactory sensitivity, these values are derived from studies using odorant diluted in a gas phase; and there appears to be a lack of reports based on the odorant presented in a fluid phase, which is the norm in a biomedical detection scenario. In the last decade, there are also no reported attempts to estimate dog olfactory detection thresholds using the more prevalent reward-based detection training methods and a standardized laboratory setting. Therefore, the aim of the present study was to estimate the olfactory detection thresholds of several dogs to amyl acetate presented in a liquid phase in such a setting.

## Materials and Methods

### Subjects

This study involved 10 detection dogs from the charity Medical Detection Dogs (UK charity registration number 1124533): 4 females and 6 males, ranging in age from 30 to 138 months (mean ± SD: 64.3 ± 38.52 months), with body weight from 10.5 kg to 24.0 kg (mean ± SD: 19.24 ± 3.97 kg), of the following breeds: Labrador Retriever (*n* = 3), Working Cocker Spaniel (*n* = 3), English Springer Spaniel (*n* = 2), and Border Collie (*n* = 2) (Table 1). These dogs were not specifically selected for their breed or type, but rather simply selected as potential working dogs by the charity.

**Table 1:** Demographic data relating to the dogs included in the study.

**Dog**	**Breed**	**Age (years)**	**Sex**
Dog 1 (Casper)	Springer Spaniel	6.10	Male, castrated
Dog 2 (Molly)	Labrador Retriever	3.4	Female, not spayed
Dog 3 (Hamish)	Working Cocker Spaniel	11.6	Male, castrated
Dog 4 (Tangle)	Working Cocker Spaniel	11	Male, castrated
Dog 5 (Sye)	Springer Spaniel	2.1	Male, castrated
Dog 6 (Amberly)	Labrador Retriever	3.5	Female, not spayed
Dog 7 (Kizzy)	Working Cocker Spaniel	3	Female, not spayed
Dog 8 (Ozzy)	Border Collie	2.6	Male, not castrated
Dog 9 (Lacey)	Border Collie	5.7	Female, spayed
Dog 10 (Chester)	Labrador Retriever	3.5	Male, castrated

This study was approved by the delegated authority of the School of Life Sciences Ethics Committee at the University of Lincoln, United Kingdom. All dogs were trained according to the ethical guidelines established by the charity Medical Detection Dogs.

### Odor Sample Preparation

The dogs were trained to detect solutions of amyl acetate (CAS 628-63-7; ≥99% Sigma Aldrich, W504009) diluted in mineral oil (Sigma Aldrich, M8410) at different concentrations. Amyl acetate was chosen on the basis of previous studies testing olfactory detection thresholds in humans (37), rodents (38, 39), and dogs (22–24). Mineral oil was used as solvent because it produces higher concentrations of volatile gases within the headspace than other potential solvents such as water (40).

A stock solution at 1:1,000 amyl acetate:mineral oil (0.5 mL amyl acetate plus 499.5 mL mineral oil) was made up to ensure consistency in the preparation of the target odor (amyl acetate concentration). A simple stepwise dilution from this stock solution was used to prepare samples with concentrations >1:1,000,000. This simple stepwise dilution consisted of 2 μL of the stock solution being mixed with an appropriate volume of mineral oil to achieve 1 mL of the desired concentration. One to three steps of 1.25-, 1.5-, and 2-fold serial dilutions of the stock solution were used to prepare target odor concentrations below 1:1,000,000. In these serial dilutions, the concentration of amyl acetate required for each step came from the diluted solution of the previous dilution step.

**Figure 1:**
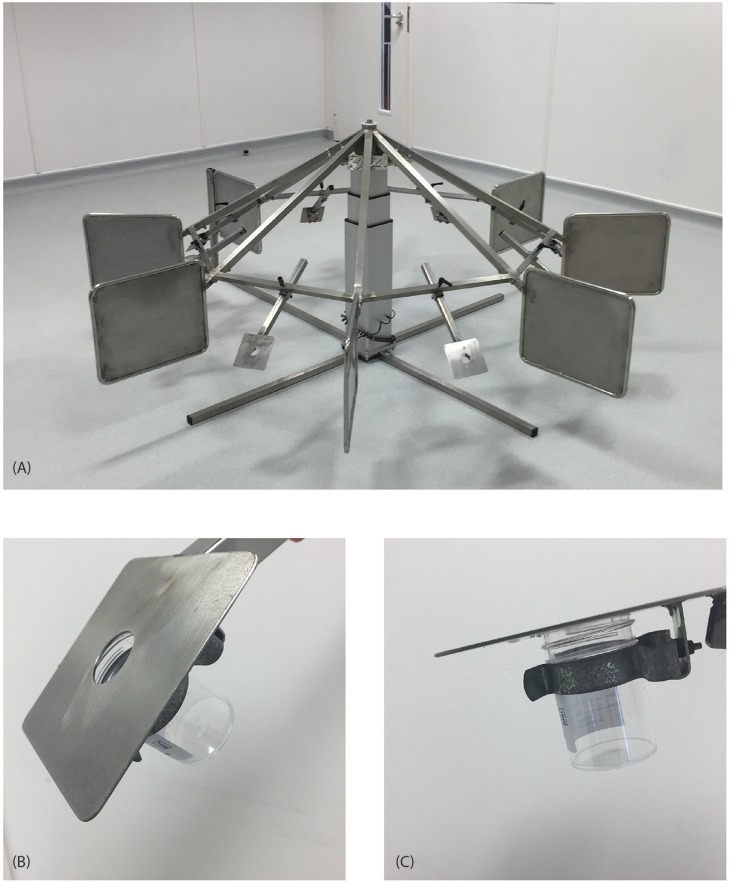
**(A)** The odor stimuli were presented using the multi-choice “carousel,” an octagonal stainless-steel stand with 8 removable arms. Each arm had a letter identified from A to H (in alphabetical order) to identify in which arm the target odor had been placed. Each position in the carousel had a number located on the base (1–8), which allowed recording the position of target odor in the carousel. **(B)** The dog sniffed the odor stimulus though a hole located in the center of the plate of the arm. **(C)** The odor stimuli were placed in a polypropylene container underneath the plate of the arm.

One milliliter of the target odor concentration was deposited in a sterile 60 mL screw-top polypropylene container (4 cm diameter, item number 360103PP; Wheaton, Rochdale, UK). Likewise, seven controls, each made up of 1 mL of mineral oil, were placed in identical sterile containers. The target odor and controls were opened and situated in an octagonal carousel (Figure 1A) similar to the circular stainless-steel odor presentation apparatus that has been used in other studies (32, 41). Each of the 8 carousel arms was removable which allowed changing of the position of the target odor on the carousel. The containers with the odor stimuli were placed underneath the plate of the arm and fixed to the arm with a metal spring clip (Figure 1C). The dogs searched for the target odor by sniffing the hole located in the center of the plate on the arm (Figure 1B).

To avoid the risk of cross contamination between controls and target odors, controls were made up first followed by the target. The target and controls were made up 10 min before the session started and set up by the researcher within the carousel. Each set of containers were used for a single session and subsequently discarded.

Similarly, a new clean set of arms was placed on the carousel for each session. The carousel was cleaned with distilled water and the set of arms washed in a dishwasher (Clasic-XX Bosch) for 45 min after each session.

For optimal estimation of the concentration of the odor stimulus, calibration curves were performed using solid phase microextraction (SPME) combined with gas chromatography-mass spectrometry (GC-MS) [Perkin Elmer Clarus 600 operated with Perkin Elmer TurboMass (2008) software] to identify the compounds and obtain direct measurement of liquid concentrations within the headspace from the stock dilution 1:1,000 amyl acetate: mineral oil (0.01 ppm) and for each 10-Fold dilution step (1:1,000; 1:10,000; 1:100,000; 1:1,000,000; 1:10,000,000; 1:100,000,000; 1:1,000,000,000). Three concentrations of amyl acetate were presented daily for each dog in a training session. Additionally, blank runs (i.e., sessions with the eight positions arms containing controls) were randomly included throughout the sessions.

### Training Procedure

The dogs worked in an indoor training room at the charity Medical Detection Dogs (see 24). During training and testing, the room was maintained at a constant temperature (~20°C) and humidity (51%).

The dogs worked with the same handler (R.H.) throughout the study to perform a “Go/No go” task. This requires the dog to issue a trained alert response in the presence of the conditioned odor (i.e., “Go” to target odor) and to withhold a response when the odor is not present (“No” go) (42).

The training involved six steps (Table 2):

*Step 1*. The dogs were classically conditioned to a clicker with food (Educ Royal Canin®) (43).
*Step 2*. A piece of tennis ball (2 cm, Head team®, yellow) was used as the initial target scent to make it easy for the dog to learn the trained alert response and use of the carousel without variations in the target odor (44, 45). Training to search the carousel was achieved by the handler presenting the dog to two carousel arms, one with a piece of tennis ball in a polypropylene sterile container and the other with an empty identical sterile container. When the dog showed interest, sniffing longer at the carousel arm with the piece of tennis ball, the dog was clicked and rewarded with Educ Royal Canin®. This was repeated until the dog reached the criterion of more than 80% correct alerting to the arm with the piece of tennis ball. Afterwards, dogs were trained to search for the piece of tennis ball in different positions on the carousel, while the remaining seven arms held empty sterile containers.
*Step 3*. After a few sessions, a conditioned alert response was introduced, so that when the dog correctly identified the position with the piece of tennis ball on the carousel, a verbal “sit” command was given to the dog by the handler, once sat, the dog was clicked and rewarded with food.
*Step 4*. The piece of tennis ball was replaced with the target odor, starting with a dilution of 1:1,000 (amyl acetate: mineral oil). The dog was clicked and rewarded with food as soon as it sniffed the target odor placed in a sterile container on the carousel. The rest of the arms contained empty sterile containers.
*Step 5*. Once the dog was able to identify and alert to the presence of the target odor with the trained alert response, controls (tubes containing mineral oil) were introduced and placed on the carousel arms to start the discrimination between the target odor and controls. The dogs had to identify either one target sample among eight samples, or ignore all the samples in a run of only control samples (a blank run). When the latter condition was introduced, the dog was recalled from the carousel once it had investigated the eight samples. In this way, the dog learned there may not always be a positive sample present and to come away from the carousel when a target was not present, positioning itself next to the handler to indicate a blank run.
*Step 6*. Detection threshold training involved the dogs working in pairs, based on their prior performance in detecting similar concentrations; each pair worked the same set of samples (target odor and controls) within a session. The order in which dogs worked (first or second) was counterbalanced during each session over different target concentrations.

**Table 2:** Training steps to teach the dog to respond to the presence of the target (amyl acetate diluted in mineral oil) and not respond to the control samples (mineral oil).

**Training phase**	
Step 1. Clicker training	- The clicker was classically conditioned to food (Educ Royal Canin®). The clicker was employed as a marker when the dog detected the target odor in the carousel.
Step 2. Training to search on the multiple-choice apparatus (“carousel”)	1. The dogs were trained to detect a piece of tennis ball (Head team®, yellow) in a sterile container. 2. The handler presented to the dog a piece of the tennis ball in a sterile container placed in the carousel arm. 3. Odor discrimination was trained between an empty sterile container and sterile container with tennis ball in different positions on the carousel.
Step 3. Introduction of trained alert response	- When the dog displayed an alert response or showed interest in the tennis ball, the handler gave a “sit” command to the dog and rewarded it.
Step 4. Training target odor (amyl acetate diluted in mineral oil)	- The piece of tennis ball was replaced with the target odor starting with a concentration of 1:1,000 (amyl acetate:mineral oil). The dog was clicked and rewarded with food as soon as the dog sniffed the target odor placed in a sterile container on the carousel. The rest of the arms remained empty.
Step 5. Detection threshold	**Stage 1—weeks 1–16** - The target dilution was presented to the dog with a systematic lowering of concentration through the stage - The handler stood next to a screen but was visible to the dogs. - The position of the target odor in the carousel was selected randomly (Excel®random number generation) and was not blind to the handler. –Screening for control samples only (searching for blanks) was also performed, where the eight positions contained only controls (i.e., the target odor was not present in the carousel). **Stage 2—weeks 17–30** - A mixture of dilutions was presented in a random fashion. - Handler was not visible to the dogs. - The position of the target in the carousel was determined by a custom-made computer target selector program and it was blind to the handler.
Step 6. Discrimination	- Once the dog was able to identify and alert to the presence of the target with the trained alert response, the controls (mineral oil) were introduced and placed on the carousel arms to discriminate between the target odor and controls. The dogs had to identify one target sample out of eight samples.
Determination of threshold criterion	Blind testing continued with serial dilutions until the proportion of true positive indications declined to consistently below 40% (4 true positives over 10 exposures to the target odor).

This detection threshold training consisted of two stages. In the first stage (weeks 1–16), target dilutions were presented to the dog with a systematic lowering of concentration. The decrease in concentration was 50% below the previous level detected by the dog, once the proportion of true positives detected by the individual at the previous concentration above 80%. During this stage, the handler stood next to a screen but was visible to the dogs. The position of the target odor in the carousel was randomly selected (Excel® random number generation) and was not blind to the handler. Blank runs were included, in which only controls were present in the apparatus.

In the second stage (weeks 17–30) a mixture of dilutions was presented in a random fashion to minimize any sample order bias. The handler stood behind the screen where he could watch the dog through a one-way mirror without being seen by the dog. The position of any target in the carousel was determined randomly using custom-made computer software, and the handler was blind with respect to the target concentration tested and the position of the target in the carousel. To reveal if the dog had alerted to the correct position, the handler pressed a keypad with the number of the carousel arm that was indicated by the dog. If the dog had indicated correctly it was clicked and rewarded.

### Structure of a Training Session

The structure of a training session has been described in detail previously by the authors [see (26)]. Each training session involved a new concentration of amyl acetate, and consisted of “runs” and “search passes”: a “run” related to the searching allowed when the target odor was in a given position on the carousel (e.g., when the odor was on arm 2); a “search pass” was a single search of arms 1–8 of the carousel. Up to three “search passes” were allowed within a “run,” with a third search pass allowed either when the dog appeared, in the handler's opinion, to show at least some hesitation on a particular carousel arm during the previous search pass or when the dog did not appear to have searched all the arms of the carousel in the previous two search passes (i.e., missed a position). A training session consisted of two changes of position of the target on the carousel per concentration (i.e., 2 “runs”).

The target and control odors were set up in the carousel by the same researcher (AC), while the dog and handler (RH) were in a separate room. The researcher left the room after setting the odor samples and entered the room between runs to change the position of the target on the carousel according to the computer program. Once the researcher left the room, the handler and the dog entered the room together and left the room between runs, but remained inside between search passes.

The session started with the handler standing next to or behind the screen (depending on training step) with the dog positioned next to him. The handler gave a verbal command to the dog to start the search. The dog sniffed the individual carousel arms without the assistance of the handler. When the dog showed the trained alert response (i.e., sit) at a position on the carousel, the handler confirmed the position through the use of key pad linked to the custom-made computer program; if the indication of the dog was correct (true positive) it was clicked, the dog left the carousel position and returned to the handler to be rewarded with food (Educ Royal Canin®). By contrast, if it was a false positive, the behavior of the dog was not reinforced (negative punishment). Blank runs (once introduced) were correctly indicated by the dog positioning itself next to the handler at the end of the run, it was clicked and rewarded as long as a false alert was not performed during the blank run.

The dogs were trained until their performance fell to below 40%, i.e., 4 true positive indications over 10 exposures to a target odor of a given concentration.

### Testing

After 30 weeks of training followed by a 7 day break, dogs were tested for their detection sensitivity. Olfactory detection threshold testing consisted of up to 3 sessions per day for 4 consecutive days for each dog. As described above, each session involved one concentration of amyl acetate. Four concentrations were chosen for each dog based on the statistical estimation of their global olfactory detection threshold trend given the individual's previous olfactory performance. Each dog was exposed 3 times to each concentration. Dogs were paired for testing within a session on the basis of similar detection threshold levels according to their previous olfactory performance, 2 dogs could not be paired (Table 3).

**Table 3:** Pairs of dogs and concentrations of amyl acetate tested for each dog, the concentrations used with each subject were determined according to the individual dog's ability as revealed in the training phase.

**Dog**	**Concentration of amyl acetate:mineral oil**
Dog 1 Dog 2	1: 1,000,000 1: 15,000,000 1: 30,000,000 1: 45,000,000
Dog 3 Dog 4	1: 10,000,000 1: 30,000,000 1: 50,000,000 1: 70,000,000
Dog 5 Dog 6	1: 10,000,000 1: 40,000,000 1: 70,000,000 1: 100,000,000
Dog 7 Dog 8	1: 10,000,000 1: 100,000,000 1: 750,000,000 1: 1,500,000,000
Dog 9	1: 10,000,000 1: 100,000,000 1: 500,000,000 1: 1,000,000,000
Dog 10	1: 1,000,000 1: 15,000,000 1: 30,000,000 1: 45,000,000

*The dogs were paired on the basis of apparently similar threshold levels during training*.

### Data Analysis

The olfactory detection performance of the dog was assessed for conformity with signal-detection theory (42, 46, 47) as follows: (1) True positive: The dog indicates the target odor in the manner in which it was trained (“sit” response), (2) True negative: The dog does not alert in the absence of the target odor, (3) False positive: The dog alerts to a non-target position (control), (4) False negative: The dog fails to exhibit the trained alert in the presence of the target odor.

To estimate the olfactory detection threshold of amyl acetate for each dog in both training and the test, a constrained logistic function was fitted to the curves describing the relationship between the proportion of true positives and amyl acetate concentration exposure to the dog. Specifically, this function was fitted using non-linear least squares, as implemented in the “minpack.lm” package for R (48) and detection thresholds estimated as the concentration at which true positives would have resulted by chance (i.e., 12.5%, 1 out of 8 possible locations). The dog's accuracy was calculated based on the number of correct assessments (true positive + true negative) over the number of all assessments (true positive + true negative + false positive + false negative) of the test data (47, 49). The accuracy of the threshold assessment was determined by how close the threshold estimation was to its true value (50). In other words, how reliable the estimation was to the actual olfactory capability revealed by the dog's ability to detect a given threshold level of amyl acetate concentration. It was predicted that low accuracy at the lowest concentration detected by the dog was a result of an increase in false negatives and false positive responses (51).

## Results

**Figure 2:**
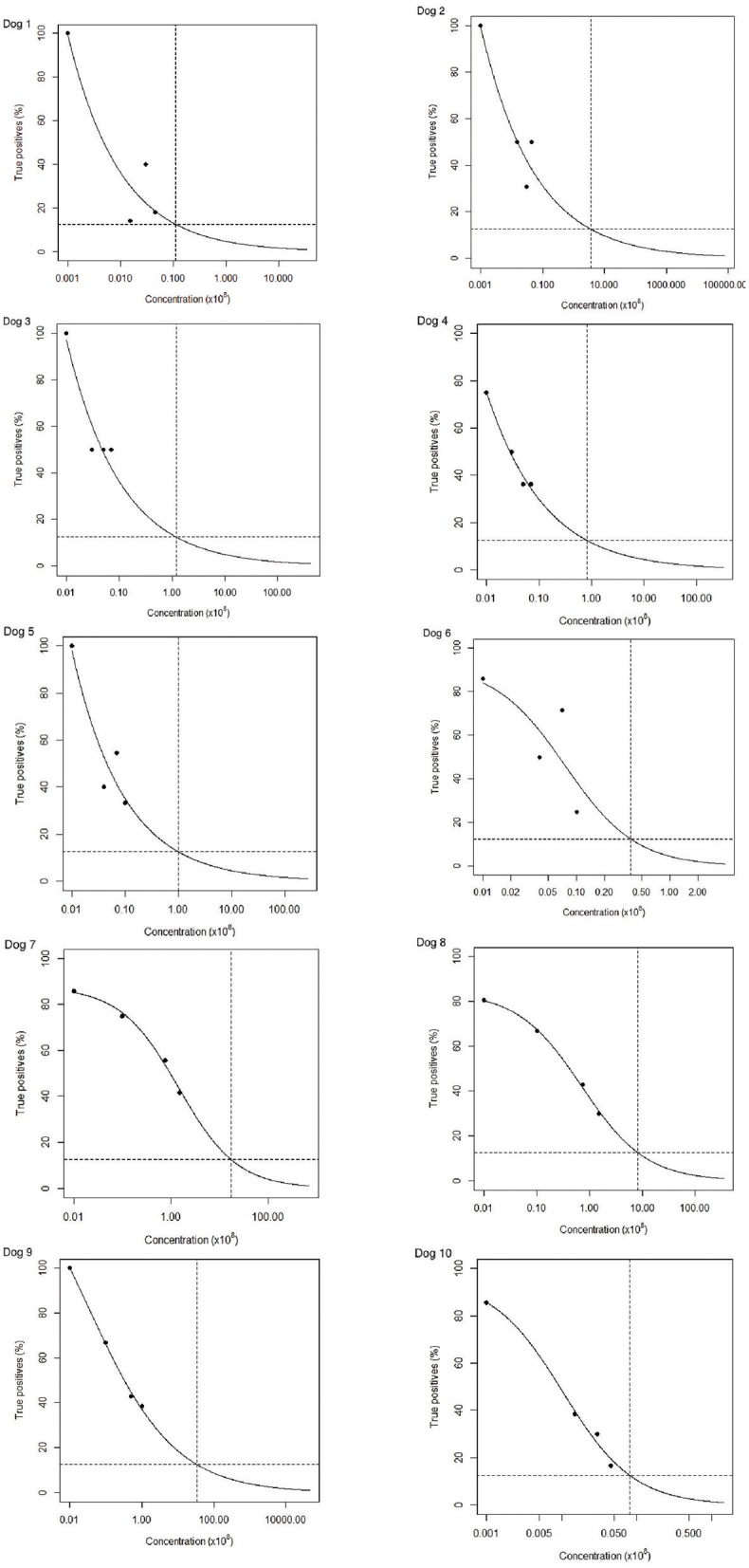
Summary of performance and estimation of thresholds of dogs 1–10 over 12 sessions of olfactory detection thresholds. Graphs show the proportion of true positives at the different concentrations of amyl acetate tested (dots) and an estimation of the global threshold trend (slope). The detection thresholds were estimated as the concentration at which the true positive rate was the equivalent of chance at 12.5% (i.e., the concentration at which the horizontal and vertical dashed lines intersect).

Olfactory detection threshold levels of amyl acetate were estimated to be between 1:40,000,000 (30 ppb) and 1:1,500,000,000 (1.5 ppt) on the basis of the fitted curve to the testing data (Figure 2, Dogs 1–10).

Accuracy measurements for the lowest concentrations detected by each dog were determined as being between 81.71 and 96.49% (Table 4). This indicates a low rate of false indications over control samples.

**Table 4:** Detection performance as a function of accuracy at the lowest concentration detected by each dog.

**Dog**	**Lowest concentration of amyl acetate: mineral oil detected**	**Accuracy (%)**
Dog 1	1: 45,000,000	81.71
Dog 2	1: 45,000,000	87.50
Dog 3	1: 70,000,000	96.49
Dog 4	1: 70,000,000	84.73
Dog 5	1: 100,000,000	90.90
Dog 6	1: 100,000,000	83.11
Dog 7	1: 1,500,000,000	87.05
Dog 8	1: 1,500,000,000	92.86
Dog 9	1: 1,000,000,000	83.33
Dog 10	1: 45,000,000	86.59

*The accuracy was calculated as the proportion of correct assessments (true positive + true negative) over total number of assessment (true positive + true negative + false positive + false negative) of the test data (47)*.

## Discussion

Any attempt to quantify odor detection and discrimination needs to consider the simplest measure of the individual's olfactory performance limits: odor threshold concentration (11, 16, 37). Below this limit, the physical stimulus is subliminal or not detectable (52). Olfactory detection thresholds vary for different chemicals and compounds and different individuals may have different thresholds for the same odorant (37). Variation in threshold performance may be influenced by genetic polymorphism of olfactory receptors (53, 54), the proportion of functional against non-functional genes (55, 56), the individual's ability to focus on searching (57), a temperament suitable for the high demands of detection training (58, 59), individual learning abilities (46, 60) and motivation (61, 62). These factors may have different degrees of impact on olfactory detection performance, which is reflected in inter-dog variability in detection thresholds to amyl acetate estimated in this study, irrespective of the fact that the dogs were trained under the same conditions. Although it was not part of this study to investigate how these factors influence the olfactory detection performance, we believe that it is possible that perceptual learning may have played an important role in the lower levels of detection thresholds observed at the end of the training and testing period (37, 63). This is in line with many studies on olfactory perceptual learning that demonstrate that the more an animal is trained to detect an odorant, the easier it is to separate that odorant from background odors (12, 64). Thus, repeated exposure is an important factor in developing olfactory sensitivity (24, 39, 65, 66) contributing to an improvement in odor acuity (63) so that the individual is able to detect at much lower thresholds than during the initial training (20, 63, 65–70).

However, as reported by Walker et al. (24) training dogs for threshold testing tasks consumes a great deal of time. For instance, one can spend approximately 6 months training two dogs for an olfactory threshold task. Likewise, this study involved 30 weeks of training for 10 detection dogs.

Previous studies assessing olfactory sensitivity in dogs have been performed using custom-fabricated devices to present odor stimuli in a standardized controlled manner (i.e., automated air stream olfactometer and test chamber) for the integration of an optimum odorant stimulus (71). However, an olfactometer controls the amount of odorant delivered to the dogs but does not necessarily facilitate effective transport of the odorant molecules into the nose. Dogs actively sniff to acquire an odor sample even when a flowing stream has been used, thus dogs dynamically control the access of odorants to the nose through sniffing (72–74) regardless of the method chosen for odor stimulus presentation (i.e., air flow or into a jar). Moreover, these laboratory measures to improve precision are not easy to reproduce. In our study, some of the dogs reached detection levels to amyl acetate at parts per trillions (ppt), yielding thresholds approximately 30-fold lower than that reported in previous work (22). This suggests that the presentation of the odor stimulus in a liquid phase using serial dilution steps provides a convenient and replicable alternative for quantifying concentrations to assess olfactory thresholds.

This study also showed that dogs achieved a high level of accuracy at the lowest threshold concentrations detected. Accuracy is used to determine how well a measure, such as olfactory detection threshold, matches the event that the test is intended to obtain, such as the actual ability of the dog to detect the target odor. Lowering the detection stimulus may produce less accurate responses due to an increase in the number of false negative and false positive responses. For instance, in the current study, the solvent (mineral oil) used in the binary mixture (i.e., amyl acetate diluted in mineral oil) was also the control (negative sample) and therefore, the dogs could be falsely responding to a similar component in the mixture at the lowest concentrations of the target odor.

Thus, the apparent difference in olfactory detection thresholds could simply reflect different tradeoffs between false and true responses and not necessarily indicate real differences in the olfactory capabilities of the dogs (51). It might be argued that, ideally, olfactory detection accuracy in dogs should be close to 100% if it is to truly reflect the dog's capabilities (60). In the present study, the accuracy was determined to be over 81.71%. Similar rates over 80% have been found for different target odors involving accelerant detection, cadaver search, and explosive detection (12, 60, 75, 76).

Several studies on scent detection dogs in the diagnosis of human disease have been reported, providing evidence for using dogs as a viable non-invasive biomedical screening method. In this biomedical detection scenario, dogs are able to detect volatile organic compounds released into body fluids such as blood and urine as a consequence of human diseases (31, 34–36). Although these VOCs are in the detection range of the dogs' olfactory sensitivity demonstrated in previous studies using odorant diluted in a gas phase, to the best of our knowledge, our study is the first investigating detection thresholds in odorants presented in a fluid phase as occurs in a biomedical detection scenario. Nevertheless, the binary mixture of amyl acetate diluted in mineral oil tested in our study only contain one hundred volatile compounds identified through the analysis using with the solid phase microextraction (SPME) combined with gas chromatography-mass spectrometry (GC-MS). By contrast, human fluids samples, such as urine, contain over seven hundred VOCs, which are present in very low concentrations and with a range of volatilities in the headspace gas (77).

Further investigation is needed to examine dogs' olfactory sensitivity to a wider range of odor stimuli, such as simple and complex odor mixtures, that would help us to better understand how dogs use their olfactory skills and strategies to optimize detection of volatile compounds within human biofluids.

## Conclusion

The first major practical contribution of the present study is that it provides much needed data on olfactory detection thresholds to amyl acetate, which is widely used in olfactory studies in dogs. This information is important given that the only other comparable study reported data for only two dogs and dates back more than 10 years. Additionally, detection thresholds reached, and accuracy level determined in our study using the olfactory stimulus presented in liquid phase evidence a reproducible alternative method to assess olfactory function in dogs.

The inter-dog variability in detection thresholds performance estimated in this study brings attention to how factors inherent to the individual (e.g., olfactory capabilities, performance and personality traits, perceptual learning abilities) can influence olfactory detection performance and the need for further investigation of these so that dogs can achieve their potential. Future studies should assess the range of factors, which may influence olfactory sensitivity in dogs and investigate dog's olfactory sensitivity in a range of odor stimuli, such as simple and complex odor mixtures.

## Author Contributions

All the authors listed have made a substantial, direct and intellectual contribution to the work, and approved for publication. The idea for the paper was conceived by AC, CG, DM, AF, HZ, RH, and TP. The study was part of Dr. Concha's Ph.D. dissertation. This work was designed by AC with guidance from DM, HZ, TP, AF, and CG. Data collection was performed by AC, RH, and CG, and data were analyzed by AC with guidance from TP, DM, and HZ. This article was primarily written by AC and the co-authors were involved with various phases of editing this article. All authors have approved the final article for submission.

### Conflict of Interest Statement

Royal Canin SAS supported the study financially; they approved the experimental design, financially supported AC, and provided the food and Edu®; food treats for the dogs used in this research. AF is employed by Royal Canin SAS. This author and co-authors have neither patent nor stock ownership, which would affect this research or publication nor do they have any membership of a company board of directors, membership of an advisory board or committee for the company.
